# Identification and Characterization of Integron-Mediated Antibiotic Resistance in the Phytopathogen *Xanthomonas oryzae* pv. *oryzae*

**DOI:** 10.1371/journal.pone.0055962

**Published:** 2013-02-21

**Authors:** Ying Xu, Qing-quan Luo, Ming-guo Zhou

**Affiliations:** 1 College of Plant Protection, Nanjing Agricultural University, Nanjing, China; 2 Shanghai Landscape Gardening Research Institute, Shanghai, China; Universidad Pública de Navarra, Spain

## Abstract

Four streptomycin-resistant isolates of *Xanthomonas oryzae* pv. *oryzae* (YNA7-1, YNA10-2, YNA11-2, and YNA12-2) were examined via PCR amplification for the presence of class 1, class 2, and class 3 integrons and *aadA1* and *aadA2* genes, which confer resistance to streptomycin and spectinomycin. The class 1 integrase gene *intI1* and the aminoglycoside adenylyltransferase gene *aadA1* were identified in all four resistant isolates but not in 25 sensitive isolates. PCR amplifications showed that 7790-bp, 7162-bp, 7790-bp, and 7240-bp resistance integrons with transposition gene modules (*tni* module) in 3′ conserved segments existed in YNA7-1, YNA10-2, YNA11-2, and YNA12-2, respectively. Subsequent analysis of sequences indicated that the integrons of YNA7-1 and YNA11-2 carried three gene cassettes in the order |*aacA3*|*arr3*|*aadA1*|. The integron of YNA10-2 carried only |*arr3*|*aadA1*| gene cassettes. The integron of YNA12-2 lacked a 550-bp sequence including part of *intI1* but it still carried |*aacA3*|*arr3*|*aadA1*| gene cassettes. The analysis of inactive mutants and complementation tests confirmed that the *aacA3* gene conferred resistance to tobramycin, kanamycin, gentamicin and netilmicin; the *arr3* gene conferred resistance to rifampicin; and the *aadA1* gene conferred resistance to streptomycin and spectinomycin. The resistance phenotypes of the four isolates corresponded with their resistance gene cassettes, except that YNA7-1 and YNA12-2 did not show rifampicin resistance. Sequence comparison revealed that no gene cassette array in GenBank was in the same order as in the integrons of the four resistant isolates in this study and the *aadA1*, which was identical in the four resistant isolates, showed 99% identity with *aadA1* sequences in GenBank. The result of a stability test showed that the resistance phenotype, the *aadA1* gene, and the *intI1* gene were completely stable in YNA7-1 and YNA12-2 but unstable in YNA10-2 and YNA11-2. To our knowledge, this is the first report of resistance integron in a phytopathogenic bacteria.

## Introduction

Bacterial blight of rice, caused by *Xanthomonas oryzae* pv. *oryzae*, is a serious disease in many rice-growing regions of the world, including the south of China [1]–[3]. Bacterial blight causes at least 10% yield loss on susceptible rice varieties when the weather is conducive [3], [4].

Although the planting of resistant rice cultivars is the main approach for controlling this disease [1], [3], application of streptomycin and other bactericides remains an important control method to complement the use of resistant cultivars and to reduce the emergence of resistance-breaking races [5]. Bismerthiazol is the most commonly used bactericide for control of bacterial blight of rice in China [5], but bismerthiazol rapidly selects for bismerthiazol-resistant strains of *X. oryzae* pv. *oryzae* [6], [7]. Streptomycin, an aminoglycoside antibiotic that has been widely used in treatment of bacterial diseases of humans and animals [8], is also used to control bacterial blight of rice in China [9].

Streptomycin has been used in agriculture in China for about 40 years [10] and has been used to prevent bacterial blight of rice for about 20 years [9]. In 2007 and 2008, 534 single-colony isolates of *X. oryzae* pv. *oryzae* were collected in the south of China to determine their susceptibility to streptomycin. The test results showed that four isolates (0.75% of the total) of *X. oryzae* pv. *oryzae* from the same county in Yunnan Province were highly resistant to streptomycin and that the resistance mechanism could not be attributed to the occurrence of *strA-strB* genes or to the *rpsL* gene mutation previously determined to cause streptomycin resistance in phytopathogenic bacteria [11]–[16].

Resistance integrons (mobile integrons) have been known to play important roles in acquisition and dissemination of antibiotic resistance genes [17]–[19]. Integrons are bacterial genetic elements that incorporate exogenous open reading frames (ORFs) by site-specific recombination and convert them to functional genes [20], [21]. Integrons consist of 5′ and 3′ conserved segments (CS) flanking a central region containing gene cassettes. The 5′ conserved region encodes three important characteristics of an integron, which are the gene for an integrase (*int*), a specific recombination site (*attI*) and a promoter [17], [20]. There are two major groups of integrons “chromosomal integrons” (also called superintegron) and “mobile integrons” (also called resistance integron) [17], [20]. In resistance integron, the exogenous genes are usually resistance genes encoding resistance to specific antibiotics. Most resistance integrons are class 1 integrons, which have been reported in many gram-negative bacteria [17], [18], [22]. The *aadA* gene encoding an aminoglycoside adenylyltransferase inactivating streptomycin and spectinomycin [23], [24], which is among the most prevalent gene in resistance integrons [23], [25], has been detected in many gram-negative bacteria isolated from humans, animals, animal food products, soil [26]–[29], and even phylloplane bacteria [30], but no *aadA* gene or resistance integron containing any other resistance gene cassette has been reported in any phytopathogenic bacteria. Here, we examined *aadA* genes and *int* genes in streptomycin-resistant and -sensitive isolates of *X. oryzae* pv. *oryzae* by PCR amplification and found all four resistant isolates carried a class 1 integron that contained an *aadA1* gene cassette. In this study, the antibiotic susceptibility profiles of the four isolates were examined and the resistance integrons were sequenced and analyzed. That the resistance genes in integron conferred resistance to the corresponding antibiotics was confirmed by both constructing inactive mutants and complementation tests. The stability of resistance and integrase genes was assessed.

## Materials and Methods

### Bacterial Strains

Bacterial strains and plasmids used in this paper are listed in Table 1. YNA7-1, YNA10-2, YNA11-2, and YNA12-2 are streptomycin-resistant and were isolated from the same county in Yunnan Province in 2007 [11]. Twenty-four isolates including YNA11-1 and YNA22-2 are streptomycin-sensitive and were randomly selected from 413 streptomycin-sensitive isolates from six provinces in the south of China in 2007, ZJ173 and PXO99 are streptomycin-sensitive isolates maintained in our laboratory [11].

**Table 1:** Bacterial strains and plasmids.

Strain or plasmid	Relevant characteristics^a^	Source or reference
Strain		
*X. oryzae* pv. *oryzae*		
YNA7-1	Wild-type resistant isolate, containing resistance integron	Lab collection
YNA10-2	Wild-type resistant isolate, containing resistance integron	Lab collection
YNA11-2	Wild-type resistant isolate, containing resistance integron	Lab collection
YNA12-2	Wild-type resistant isolate, containing resistance integron	Lab collection
YNA11-1	Wild-type sensitive isolate	Lab collection
YNA22-2	Wild-type sensitive isolate	Lab collection
ZJ173	Wild-type sensitive isolate	Lab collection
PXO99	Wild-type sensitive isolate	Lab collection
PXO1-1	PXO99 with pUFRaacA3, Km^r^, Gm^r^/Tob^r^/Ntl^r^	this study
PXO2-1	PXO99 with pUFRarr3, Km^r^, Rif^r^	this study
PXO3-1	PXO99 with pUFRaadA1, Km^r^, Sm^r^/Sp^r^	this study
PXO4-1	PXO99 with pUFRintegron, Km^r^, Gm^r^/Tob^r^/Ntl^r^, Rif^r^, Sm^r^/Sp^r^	this study
MaacA3	*aacA3* Mutant of YNA11-2, constructed through homologous recombination,Amp^r^, Km^s^/Gm^s^/Tob^s^/Ntl^s^	this study
Marr3	*arr3* Mutant of YNA11-2, constructed through homologous recombination,Amp^r^, Rif^s^	this study
MaadA1	*aadA1* Mutant of YNA11-2, constructed through homologous recombination,Amp^r^, Sm^s^/Sp^s^	this study
*Escherichia coli*		
DH5α	F^−^,φ80d *recA lacZ* △M15	Takara
Plasmids		
pMD18-T	Amp^r^, *lac*Z, T-easy vector for ligation homologous fragments through PCR	Takara
pUFR034	Km^r^, *IncW*, *Mob^+^*, *mob(p)*, *LacZa^+^*, PK2 replicon, cosmid	[46]
pUFRintegron	A fragment containing the ORF of *aac*A3, *arr*3, *aadA1* gene cloned into pUFR034	this study
pUFRaacA3	an *aacA3* gene ORF cloned into pUFR034	this study
pUFRarr3	an *arr*3 gene ORF cloned into pUFR034	this study
pUFRaadA1	an *aadA1* gene ORF cloned into pUFR034	this study

a Sm, streptomycin; Km, kanamycin; Gm, gentamicin; Amp, ampicillin; Rif, rifampicin; Tob, tobramycin; Ntl, netilmicin; Sp, spectinomycin; r indicates resistance to the antibiotic; s indicates susceptibility to the antibiotic.

### Bacterial DNA Preparation, PCR, and Nucleotide Sequencing

Template DNA was prepared using AxyPrep™ Bacterial Genomic DNA Miniprep Kit (Axygen, China). Isolates were initially characterized for streptomycin resistance-related genes including the *aadA1* and *aadA2* gene and integrase genes including the *intI1*, *intI2*, and *intI3* gene by PCR. The 5′ conserved segments of the integron was characterized using primers IRI, the 3′ conserved segments was characterized using primers IRT and primer pairs for genes *qac△E1* and *sul1*. The complete sequence of the integron was cloned with primer pairs TniR-a and IRI, TniaR and TniA. To amplify chromosomal integrons in *X. oryzae* pv. *oryzae*, a pair of primers ilvd-f and intergon-r was designed according to the genome sequence of *X. oryzae* pv. *oryzae* KACC10331 (GenBank accession No. AE013598). When the length of target products was shorter than 1000 bp, the 50 µl PCR mixture consisted of 2 µl of template, 5 µl of 10×PCR buffer Mg^2+^ Free (TaKaRa Taq^Tm^, TaKaRa, China), 1.25 units of Taq polymerase (TaKaRa Taq^Tm^, TaKaRa, China), 0.4 µM of each primer, 1.5 mM MgCl_2_, and 200 µM of each dNTP. When the length of target products was longer than 1000 bp, the 50 µl PCR mixture consisted of 2 µl of template, 5 µl of 10×PCR buffer Mg^2+^ Free (TaKaRa LA PCR Buffer II, TaKaRa, China), 2.5 units of Taq polymerase (TaKaRa LA Taq, TaKaRa, China), 0.4 µM of each primer, 2.5 mM MgCl_2_, and 400 µM of each dNTP. PCR was performed in a TaKaRa PCR Thermal Cycler Dice (TaKaRa, China). Primer pairs and conditions for amplifying were listed in Table S1 [31]. *Escherichia coli* JM109/pHM1 containing the *aadA1* and the *intI1* genes was used as a positive control when *intI1* and *aadA1* were amplified. Amplification products were analyzed by agarose gel electrophoresis. Amplicons were purified with the AxyPrep™ PCR cleanup kit (Axygen, USA), and the purified products were sequenced by Shanghai Sangon (Sangon, China). If necessary, amplicons were individually excised from the agarose gel and purified using the Axygen gel extraction kit (Axygen, USA) according to the manufacturer’s instructions. Following purification, the PCR products were ligated to a pMD19-T Vector (TaKaRa, China) according to the manufacturer’s recommendations and sequenced by Shanghai Sangon (Sangon, China).

### Nucleotide Sequence Analysis and Accession Number

Nucleotide sequence analysis was performed by BLAST (National Center for Biotechnology Information [NCBI]) and BioEdit 7.0 (http://www.mbio.ncsu.edu/BioEdit/bioedit.html). The nucleotide sequences of the resistance integrons of streptomycin-resistant isolates YNA7-1, YNA10-2, YNA11-2, and YNA12-2 were submitted to GenBank and assigned accession numbers HQ662554, HQ662555, HQ662556, and HQ662557, respectively. The nucleotide sequence of the chromosomal integrase and its flanking region from YNA11-2 was submitted to GenBank and assigned accession numbers JX998161. The accession number of the chromosomal integrase and its flanking region from ZJ173 is JX998162.

### Antibiotic Susceptibility

The antibiotic susceptibility profiles of *X. oryzae* pv. *oryzae* isolates were first examined by the paper disk diffusion method, and the results were confirmed by the minimum inhibitory concentration (MIC) method. The paper disk diffusion method was carried out on Mueller–Hinton agar with 15 different antibiotic disks (Tianhe, China) according to the guidelines of the Clinical and Laboratory Standards Institute [32]. The 15 different antibiotics were tobramycin (10 µg per disk), kanamycin (30 µg per disk), rifampicin (5 µg per disk), netilmicin (30 µg per disk), gentamicin (10 µg per disk), amikacin (30 µg per disk), ampicillin (10 µg per disk), cephalothin (30 µg per disk), chloramphenicol (30 µg per disk), nitrofurantoin (300 µg per disk), neomycin (30 µg per disk), novobiocin (5 µg per disk), oxacillin (1 µg per disk), polymyxin B (300 IU per disk), and vancomycin (30 µg per disk). *Escherichia coli* ATTC 25922 was used as a quality control strain for the antibiotic disk diffusion method. The quality control ranges for ATTC 25922 followed the CLSI guidelines [33], while the antibiotic susceptibility results of measured isolates were determined through comparison among themselves for lack of an interpretive standard for *X. oryzae* pv. *oryzae*.

MICs were determined with a slightly modified agar dilution method [15]. Bacterial suspension in the late logarithmic growth phase was diluted to about 10^7^ CFU/ml, and 5 µl of the suspension was pipetted onto nutrient agar (NA) plates separately containing serial concentrations of streptomycin, spectinomycin, kanamycin sulfate, tobramycin, gentamicin sulfate, rifampicin, or netilmicin. All of these except netilmicin, which was obtained from Shanghai Asia Pioneer Pharmaceutical Co., Ltd. (Shanghai, China), were obtained from Bio Basic Inc. (Markham, Canada). The concentrations of antibiotic used in the MIC test included 1, 5, 10, 50, and 100 µg/ml and one additional concentration for spectinomycin (500 µg/ml), two additional concentration for streptomycin (200 and 300 µg/ml) and two additional concentrations for rifampicin (0.1 and 0.5 µg/ml). The plates were incubated at 28°C for 72 h before they were examined for bacterial growth. The lowest concentration that completely inhibited bacterial growth was considered the MIC value.

### Construction and Analysis of Resistance Gene Mutants

To reveal the contribution of the three genes (*aacA3*, *arr3* and *aadA1*) in gene cassettes to antibiotic resistance, target inserted mutants were constructed through homologous single recombination [34]. To construct an *aacA3* mutant, *arr3* mutant and *aadA1* mutant in *X. oryzae* pv. *oryzae*, a 348-bp fragment containing a partial *aacA3* coding region, a 324-bp fragment containing a partial *arr3* coding region, and a 355-bp fragment containing a partial *aadA1* coding region were amplified through PCR from the isolate YNA11-2, then the three fragments were ligated to vector pMD18-T (TaKaRa, China), respectively. Ligation and transformation were conducted according to the manufacturer’s instructions. The fragments of PCR amplification were analyzed by agarose gel electrophoresis and the sequences of the fragments and their flanking regions in pMD18-T were identified by DNA sequencing. The three recombinant plasmids were electroporated independently into resistant isolate YNA11-2. The preparation of electrocompetent cell of YNA11-2 and electroporation performed on electroporation instrument Eppendorf Mutiporator (Eppendorf, Germany) were as described [35], except the medium was NB instead of SOC. Transformants were selected on NA plates with 100 µg/ml ampicillin. To verify transformants were correctly inserted mutants through homologous single recombination, each putative mutant was tested through PCR with two primer pairs corresponding to the inserted sequence and its flanking region (Table S1). The amplicons were analyzed by agarose gel electrophoresis and were sequenced to verify the mutants were correctly inserted. The primer pairs and conditions for amplification were listed in Table S1 and the components of PCR were as mentioned previously. The PCR amplifications were carried out in a PTC 200 thermocycler (MJ Research, Inc.). The MIC of verified mutants to antibiotics including streptomycin, spectinomycin, kanamycin sulfate, gentamicin sulfate, tobramycin, netilmicin and rifampicin were evaluated.

### Function Complementation of the Gene *aacA3*, *arr3* and *aadA1* in Gene Cassettes

To further elucidate the function of the three genes (*aacA3*, *arr3* and *aadA1*) in gene cassettes to antibiotic resistance, three segments of the *aacA3*, *arr3*, *aadA1* gene and a segment of integron containing all three genes *aacA3*, *arr3*, *aadA1*, were separately cloned to a vector and transferred to PXO99, a sensitive isolate of *X. oryzae* pv. *oryzae*. Three pairs of primers were designed to amplify the gene *aacA3*, *arr*3, *aadA1* respectively from YNA11-2 and the primer pair integronf and integronr was designed to amplify a fragment containing all three resistance gene (*aacA3*, *arr3* and *aadA1* gene) (Table S1). The primer pairs and conditions for amplification were listed in Table S1 and the components of PCR were as mentioned previously. The PCR amplifications were carried out in a PTC 200 thermocycler (MJ Research, Inc.). Amplification products were analyzed by agarose gel electrophoresis and sequenced by Shanghai Sangon (Sangon, China).

In order to construct recombinant plasmid, restriction enzyme site and protective bases pairs were added in the 5′ terminal of primer when the primers were designed (Table S1). Plasmid pUFR034 purified with the AxyPrep™ Plasmid Miniprep Kit (Axygen, USA), and amplicons from the four primer pairs purified with the AxyPrep™ PCR cleanup kit (Axygen, USA) were double digested with *Sac*I and *Kpn*I respectively. After that, amplicons were ligated to the vector pUFR034 using T4 DNA ligase (Takara, China). Four recombinant plasmids containing the gene *aacA3*, *arr3*, *aadA1* and all three were constructed respectively (designated plasmid pUFRaacA3, pUFRarr3, pUFRaadA1 and pUFRintegron, Table 1).

The four recombinant plasmids pUFRaacA3, pUFRarr3, pUFRaadA1 and pUFRintegron were electroporated into PXO99, respectively. The preparation of electrocompetent cells of PXO99 and electroporation were performed as described in previous section. The transformants were selected on NA plates containing 20 µg/ml kanamycin. Positive transformants were screened by PCR of target genes and further confirmed by double digestion with *Sac*I and *Kpn*I of recombinant plasmids. Finally, the MIC of positive transformants to streptomycin, spectinomycin, kanamycin sulfate, gentamicin sulfate, tobramycin, netilmicin and rifampicin were examined.

### Stability Assay of the Resistance Integrons

The stability assay of the resistance integrons was performed through consecutive transfers on plates without antibiotic pressure described by Cox *et al*. [36]. The four resistant isolates (YNA7-1, YNA10-2, YNA11-2, and YNA12-2) harboring resistance integrons were initially transferred from stock cultures to antibiotic-free NA plates to obtain actively growing culture. One colony of each isolate was transferred to a fresh NA plate, which was incubated for 2 days at 28°C to be used as the first transfer. The bacteria on this plate were then streaked onto a fresh NA plate and incubated for 2 days at 28°C. A total of 20 successive transfers were made on NA plates without antibiotics. A full loop of bacteria needs to be used for each transfer so that the characteristic of the bacteria streaked onto the fresh plate could represent the bacteria on the source plate.

After the transferred colonies had grown on NA without antibiotic, a loop of bacteria was transferred to a fresh NA plate by plate streaking in order to obtain single colonies. Among the single colonies growing on this plate, 20 single colonies were selected for testing. The simplified MIC method with 50 µg/ml streptomycin was used to determine susceptibility to streptomycin [11]. The *intI1* and *aadA1* gene were detected by PCR as described previously except the preparation of template DNA was as described by Xu *et al*. [11] and 10 µl of template DNA was used instead of 2 µl of template DNA in 50 µl PCR mixture. This experiment was performed twice. In the first trial, the tests were performed on the 10th and 20th transfer. In the second trial, the tests were performed on the 5th, 10th, 15th, and 20th transfer.

## Results

### Detection of Resistance Integrons that Contain the *aadA1* Gene Cassette Conferring Streptomycin Resistance

PCR-based examination for the presence of an integron was carried out on the four streptomycin-resistant isolates (YNA7-1, YNA10-2, YNA11-2, and YNA12-2) and three streptomycin-sensitive isolates (ZJ173, YNA11-1, and YNA22-2). The PCR amplification used oligonucleotide primers specific for three integrase genes (*int*), which indicate the presence of an integron, and for two adenyltranseferase genes (*aadA*), which confer resistance to streptomycin. No amplification products were obtained from any of these isolates when the primers specific for *intI2*, *intI3*, or *aadA2* were used (Table 2). PCR with primers specific for *intI1* and *aadA1* produced amplicons from the four resistant isolates but not from the three sensitive isolates (Table 2). To confirm the result, 22 additional sensitive isolates were screened for the presence of the *intI1* gene and the *aadA1* gene. In agreement with the negative results shown for the three sensitive isolates in Table 2, none of the 22 additional sensitive isolates showed any amplification products (data not shown), which indicated that the *aadA1* gene in the class 1 integron might contribute to the streptomycin resistance in the four resistant isolates.

**Table 2:** PCR amplification of integron-related genes from streptomycin-sensitive and -resistant isolates of *X. oryzae* pv. *oryzae* a.

Isolate	Streptomycin susceptibility^b^	Gene or primer pair											
		*intI1*	*intI2*	*intI3*	*aadA1*	*aadA2*	IRI andaadA1-R	*qac△E1*	*sul1*	intI-Rand IRT	IRI and TniR-a	*intIA*	
YNA7-1	R	+	−	−	+	−	+	−	−	−	+	+	
YNA10-2	R	+	−	−	+	−	+	−	−	−	+	+	
YNA11-2	R	+	−	−	+	−	+	−	−	−	+	+	
YNA12-2	R	+	−	−	+	−	+	−	−	−	+	+	
ZJ173	S	−	−	−	−	−	ND	ND	ND	ND	ND	+	
YNA11-1	S	−	−	−	−	−	ND	ND	ND	ND	ND	+	
YNA22-2	S	−	−	−	−	−	ND	ND	ND	ND	ND	+	

a + and − indicate positive and negative PCR amplification; ND indicates not determined.

b R indicates resistance to streptomycin and S indicates susceptibility to streptomycin.

### Resistance Integron Sequences

**Figure 1:**
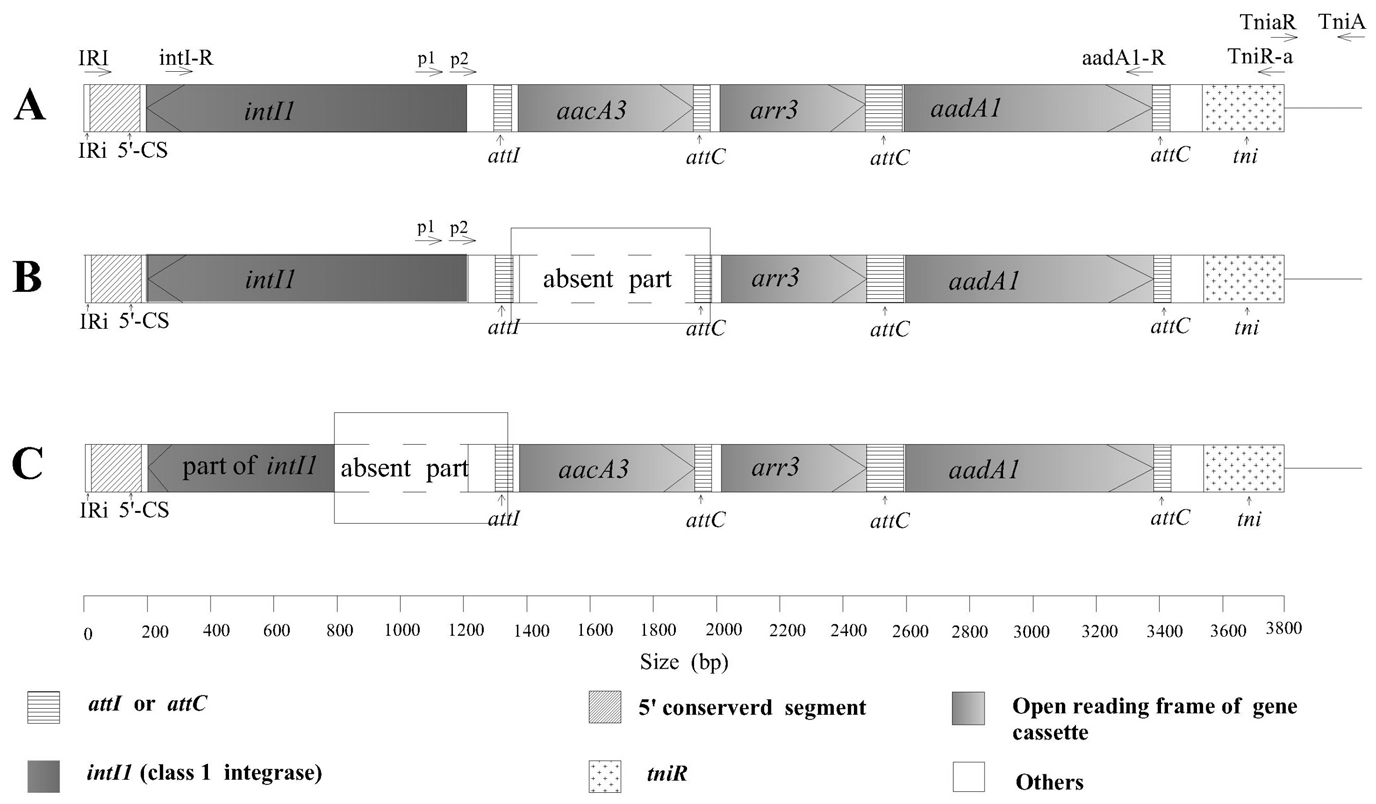
Structural organization of integrons in four resistant isolates of *Xanthomonas oryzae* pv. *oryzae*. A. YNA7-1 and YNA11-2; B. YNA10-2; C. YNA12-2. The complete sequences of the resistance integrons in the four resistant isolates through PCR with primer pairs TniR-a and IRI, TniaR and TniA. The resistance integrons in YNA7-1, YNA10-2, YNA11-2, and YNA12-2 contained 7790, 7162, 7790, and 7240 bp, respectively. The sequence of *tni* module was long and not completely shown in this figure. The sequences of the resistance integrons of YNA7-1 and YNA11-2 were identical. Compared with YNA7-1 and YNA11-2, the sequence from position 1350 to 1977 was absent in the resistance integron of YNA10-2, and the sequence from 791 to 1340 was absent in the resistance integron of YNA12-2. Location of some primers used for PCR is shown in the figure. P1 and p2 are promoter areas of integrons.

For further characterization of these integrons, the four isolates carrying the class 1 integron were screened for the *qac△E1* gene and the *sul1* gene, which are part of the 3′ conserved segment (CS), and for the 5′ and 3′ ends of the inverted repeat of the integron (Table 2). When amplifying the 3′ CS including *qac△E1*, *sul1*, and 3′ inverted repeats (using primer IRT) of the class 1 integron, PCR did not yield any products with the four resistant isolates, which indicated that the 3′ CS that existed in most integrons was absent in the four resistant isolates (Table 2). Positive amplification results from resistant isolates with primers aadA1-R and IRI, which are specific for the 5′ end of the inverted repeat of class 1 integrons, indicated that the 5′ CS existed in the four resistant isolates. Post *et al.* [37] reported that some class 1 integrons contained a transposition gene module (*tni* module) of Tn*402* instead of *qac△E1* and *sul1* in the 3′ CS, the primer TniR-a (designed according to the sequence of the *tni* gene in the 3′ CS, accession number X72585) and the primer IRI in the 5′ CS were used to amplify the sequence of the resistance integron. The amplification results for the four resistant isolates using this primer pair were all positive (Figure 1, Table 2), which indicated that the 3′ CSs of the four resistant isolates were *tni* modules instead of the 3′ CSs that occur in most integrons. The complete *tni* module was amplified by the primer pair TniaR and TniA (Figure 1).

The complete sequences of the resistance integrons in the four resistant isolates were analyzed through comparison and BLAST in GenBank (Table 3, Table 4, and Figure 1). The resistance integrons in YNA7-1, YNA10-2, YNA11-2, and YNA12-2 contained 7790, 7162, 7790, and 7240 bp, respectively. The sequences of the resistance integrons of YNA7-1 and YNA11-2 were identical. Compared with YNA7-1 and YNA11-2, the sequence from position 1350 to 1977 was absent in the resistance integron of YNA10-2, and the sequence from 791 to 1340 was absent in the resistance integron of YNA12-2 (Table 4 and Figure 1). The cassette array in the resistance integrons of YNA7-1 and YNA11-2 was |*aacA3*|*arr3*|*aadA1*| (Table 3), in which *aacA3* theoretically conferred resistance to tobramycin, kanamycin, gentamicin, and netilmicin; *arr3* theoretically conferred resistance to rifampicin; and *aadA1* theoretically conferred resistance to streptomycin and spectinomycin. Compared to YNA7-1, YNA10-2 lacked the *aacA3* gene cassette but had the |*arr3*|*aadA1*| gene cassettes (Table 3 and Figure 1), and YNA12-2 lacked a 550-bp sequence, including part of the *intI1* gene, but still carried the |*aacA3*|*arr3*|*aadA1*| gene cassettes (Table 3 and Figure 1). The nucleotide sequences of integrons in the four resistant isolates were identical except for the absent parts.

**Table 3:** Characteristics of resistance integrons in four resistant isolates of *X. oryzae* pv. *oryzae*.

Isolate	Integron size (bp)	Array of gene cassettes	Antibiotic resistance phenotype^a^
YNA7-1	7790	|*aacA3*|*arr3*|*aadA1*|	Tob/Gm/Ntl/Km, Sm/Sp
YNA10-2	7162	|*arr3*|*aadA1*|	Rif, Sm/Sp
YNA11-2	7790	|*aacA3*|*arr3*|*aadA1*|	Tob/Gm/Ntl/Km, Rif, Sm/Sp
YNA12-2	7240	|*aacA3*|*arr3*|*aadA1*|	Tob/Gm/Ntl/Km, Sm/Sp

a Antibiotic resistance phenotype: Tob, tobramycin; Gm, gentamicin; Ntl, netilmicin; Km, kanamycin; Rif, rifampicin; Sm, streptomycin; Sp, spectinomycin.

**Table 4:** Predicted genes in resistance integrons in four resistant isolates of *X. oryzae* pv. *oryzae* and the alignment of these genes relative to those in GenBank.

Gene	Position^a^	Length (bp)	YNA10-2	YNA12-2	Identity^b^	Accession no.^c^	Description of the identical gene
Incomplete IRi	1–19	19	Same	Same	100%	AY033653.3	*Pseudomonas aeruginosa* isolate 96 plasmid pOZ176, Tn402-like class 1 integron, 5′ CS
Partial 5′CS	20–196	177	Same	Same	100%	AY033653.3	*Pseudomonas aeruginosa* isolate 96 plasmid pOZ176, Tn402-like class 1 integron, 5′ CS
*intI1*	197–1210	1014	Same	Absent 791–1340	100%	GQ293501.1	*Escherichia coli* strain IncA/C2 plasmid pRYC103T24, IntI (*intI*)
P1 promotor area	1095–1123	29	Same	Absent 791–1340	100%	GQ293501.1	*Escherichia coli* strain IncA/C2 plasmid pRYC103T24, IntI (*intI*)
P2 promotor area	1214–1239	26	Same	Absent 791–1340	100%	GQ293501.1	*Escherichia coli* strain IncA/C2 plasmid pRYC103T24, IntI (*intI*)
*attI*	1295–1352	58	Absent 1350–1977	Absent 791–1340	100%	DQ914960.2	*Pseudomonas aeruginosa* strain PA0905 class 1 integron, partial sequence
*aacA3*	1372–1926	555	Absent 1350–1977	Same	100%	EF127959.1	*Acinetobacter baumannii* isolate K43 class 1 integron Aac6-II (*aac6-II*)
*attc* for *aacA3*	1921–1980	60	Absent 1350–1977	Same	100%	EF127959.1	*Acinetobacter baumannii* isolate K43 class 1 integron Aac6-II (*aac6-II*)
*arr3*	2011–2463	453	Same	Same	100%	EU340416	*Acinetobacter baumannii* isolate 509107 class 1 integron rifampin ADP-ribosylating transferase *(arr-3*)
*attc* for *arr3*	2470–2583	114	Same	Same	100%	FM207631.1	*Aeromonas caviae* partial class 1 integron containing *aar-3* gene.
*aadA1*	2587–3378	792	Same	Same	99%	EF527229.1	*Escherichia coli* strain 55 class 1 integron, aminoglycosidase adenyltransferase (*aadA1*)
*attc* for *aadA1*	3380–3439	60	Same	Same	100%	EF527229.1	*Escherichia coli* strain 55 class 1 integron, aminoglycosidase adenyltransferase (*aadA1*)
*tni* module	3536–7790	4255	Same	Same	100%	JN983043.1	*Salmonella enterica* subsp. *enterica* serovar Heidelberg plasmid pSH111_166 tni module

a Positions were defined by the order of integron of YNA7-1 and YNA11-2.

b Maximum identity at nucleotide acid level.

c Accession no. of most similar gene in GenBank.

According to BLAST searches of the complete sequences of resistance integrons, no gene cassettes of integrons in GenBank were arrayed in the same order as those in the resistance integrons in the four *X. oryzae* pv. *oryzae* isolates reported here. So the resistance genes in the *X. oryzae* pv. *oryzae* integrons were compared one by one in GenBank. BLAST analysis (Table 4) indicated that all genes in resistance integrons in *X. oryzae* pv. *oryzae* had 100% identity with genes in GenBank except the *aadA1* gene. The *aadA1* gene in this study showed 99% identity with the *aadA1* gene of *Escherichia coli* strain 55 in the GenBank database (accession No. EF527229.1), with a substitution of G for A at position 236, which resulted in an amino acid substitution of glycine (Gly) for glutamic acid (Glu) at codon 79.

### Chromosomal Integrase Sequence

To find out the relationship between chromosomal integron and resistance integron (mobile integron) in *X. oryzae* pv. *oryzae*, chromosomal integrase and its flanking sequence were amplified with the primer pair ilvd-f and intergon-r. The 3250-bp nucleotide sequences containing the chromosomal integrase gene *intIA* and its flanking sequence were obtained from four resistant isolates and two sensitive isolates YNA11-1 and YNA22-2. Sequence analysis showed that the amplified nucleotide sequences from four resistant isolates and two sensitive isolates were 100% identical, which indicated there was no direct relationship between the chromosomal integrase and the antibiotic resistance in *X. oryzae* pv. *oryzae*. The *intIA* sequence of ZJ173, which was isolated from a different district, was 99.7% identical to the six isolates from Yunnan Province.

### Antibiotic Resistance Phenotype

The antibiotic susceptibility of the integron-carrying isolates YNA7-1, YNA10-2, YNA11-2, and YNA12-2 and the non-integron-carrying isolates ZJ173, YNA11-1, and YNA22-2 was determined by the paper disk diffusion method and the MIC method. In the paper disk diffusion tests, all seven isolates were sensitive to amikacin, ampicillin, cephalothin, chloramphenicol, neomycin, nitrofurantoin, novobiocin, polymyxin B, and vancomycin and resistant to oxacillin (resistance to oxacillin was not related to the resistance integron). Some or all of the four integron-carrying isolates were resistant to tobramycin, kanamycin, netilmicin, gentamicin, and rifampicin, while the three non-integron-carrying isolates were sensitive to these antibiotics. The MIC determination result showed that the four integron-carrying isolates were resistant to spectinomycin, while the three non-integron-carrying isolates were sensitive to spectinomycin (Table 5). In agreement with this result, the four integron-carrying isolates contained the *aadA1* gene, which encodes aminoglycoside-3′-adenylyltransferase and thereby confers resistance to both streptomycin and spectinomycin [38]. Three of the integron-carrying isolates (YNA7-1, YNA11-2, and YNA12-2) were resistant to tobramycin, kanamycin, netilmicin, and gentamicin, while one integron-carrying isolate (YNA10-2) and the three non-integron-carrying isolates were sensitive to these four antibiotics. In agreement with this result, the integron-carrying isolates YNA7-1, YNA11-2, and YNA12-2 contained the *aacA3* gene, which encodes aminoglycoside-6′-N-acetyltransferase and thereby confers resistance to tobramycin, kanamycin, netilmicin, and gentamicin [39], [40], while the *aacA3* gene was absent in the integron-carrying isolate YNA10-2 and the three non-integron-carrying isolates. Two integron-carrying isolates (YNA10-2 and YNA11-2) showed rifampicin resistance, consistent with the gene *arr3*, which encodes ADP-ribosyl transferase and thereby confers resistance to rifampicin [41]. The other two integron-carrying isolates (YNA7-1 and YNA12-2), however, also contained the *arr3* gene but were sensitive to rifampicin. In other words, there was a discrepancy between genotype and phenotype of rifampicin susceptibility in YNA7-1 and YNA12-2. In summary, the antibiotic resistance phenotypes and resistance gene cassettes were consistent for the *X. oryzae* pv. *oryzae* isolates in this study, except that isolates YNA7-1 and YNA12-2 showed rifampicin-sensitivity but carried the rifampicin-resistance gene cassette.

**Table 5:** The MIC (minimum inhibitory concentration, µg/ml) of *X. oryzae* pv. *oxyzae* isolates to diverse antibiotics.

Isolate	Antibiotic						
	Streptomycin	Spectinomycin	Tobramycin	Kanamycin	Netilmicin	Gentamicin	Rifampicin
YNA7-1	300	>500	10	100	100	100	0.1
YNA10-2	300	>500	1	1	1	1	10
YNA11-2	300	>500	10	100	100	100	10
YNA12-2	300	>500	10	100	100	50	0.1
ZJ173	1	5	1	1	1	1	0.1
YNA11-1	1	5	1	1	1	1	0.1
YNA22-2	1	1	1	1	1	1	0.1
PXO99	1	5	1	1	1	1	0.1
YNA11-2*^a^	200	>500	50	100	100	50	10
PXO1-1	ND	ND	50	>100	100	50	ND
PXO2-1	ND	ND	ND	>100	ND	ND	10
PXO3-1	100	>500	ND	>100	ND	ND	ND
PXO4-1	100	>500	50	>100	100	50	10
MaacA3	100	>500	1	1	1	1	5
Marr3	50	>500	50	50	100	50	0.5
MaadA1	1	5	50	50	100	50	10

a YNA11-2* was an isolate of YNA11-2 which has been preserved under low-temperature for three years.

### Analysis and Characterization of Resistance Gene Mutants

**Figure 2:**
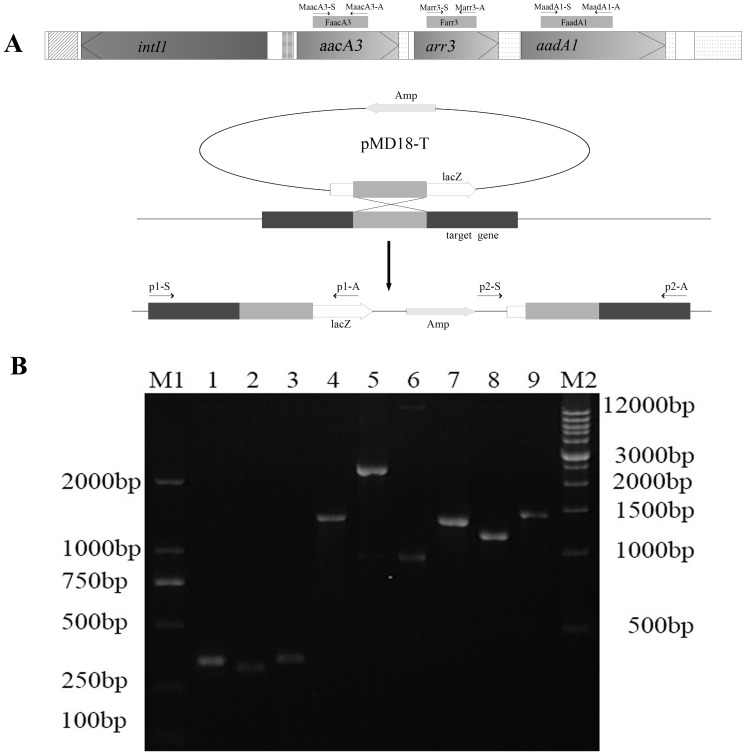
Construction and analysis of resistance gene mutants. A. Schematic representation of homologous single recombination between a target resistance gene fragment cloned in pMD18-T and the target resistance gene in YNA11-2. Location of primers used for PCR is shown in the figure. The shaded box areas represent the target resistance gene while the lightly shaded area represents a cloned resistance gene fragment where homologous recombination occurred between pMD18-T and the genome of YNA11-2. When the mutant MaacA3 was analyzed, the primer accyan1-S, accyan1-A, accyan2-S and accyan2-A were used as the p1-S, p1-A, p2-S and p2-A, respectively. When the mutant Marr3 was analyzed, the primer arryan2-S, arryan2-A, arryan4-S and arryan4-A were used as the p1-S, p1-A, p2-S and p2-A, respectively. When the mutant MaadA1 was analyzed, the primer aadayan4-S, aadayan4-A, aadayan5-S and aadayan5-A were used as the p1-S, p1-A, p2-S and p2-A, respectively. B. Electrophoresis of three resistance genes fragments and PCR identification of three mutants. M1, DNA markers; Lane 1–3, PCR products of three resistance genes fragments FaacA3, Farr3 and FaadA1; Lane 4–9, PCR products of MaacA3 with primer pairs p1 and p2, PCR products of Marr3 with primer pairs p1 and p2, PCR products of MaadA1 with primer pairs p1 and p2; M2, DNA markers.

Three resistance gene fragments including a 348-bp fragment of the *accA3* gene (designated FaccA3), a 324-bp fragment of the *arr3* gene (designated Farr3) and a 355-bp fragment of the *aadA1* gene (designated FaadA1) were respectively cloned into pMD18-T (Figure 2), and identified by sequencing (data not shown). The Vector pMD18-T is a suicide plasmid in *X. oryzae* pv. *oryzae*, selection of ampicillin-resistant transformants arising from a homologous single recombination event between the plasmid pMD18-T containing the fragment of resistance gene and the genome of YNA11-2 impelled inactivation of the target resistance gene (Figure 2). The three inserted mutants were respectively identified by PCR analysis using two corresponding primer pairs. For each primer pair, one primer was located in the inserted fragment and the other primer located outside of the inserted fragment (Figure 2). The expected size of PCR amplicons was obtained in the three inserted mutants (Figure 2), while no PCR products were detected in the control parental strain YNA11-2 (data not shown). Further sequencing of PCR products confirmed that the *aacA3* gene, the *arr3* gene and the *aadA1* gene had been mutated at the correct position (data not shown).

The MIC determination result showed that the mutant MaacA3 containing an inactivated *aacA3* gene, was sensitive to kanamycin, tobramycin, netilmicin and gentamicin, while it was still resistant to streptomycin, spectinomycin and rifampicin. The mutant Marr3 carrying an inactivated copy of gene *arr3*, was sensitive to rifampicin, while it was still resistant to streptomycin, spectinomycin, kanamycin, tobramycin, netilmicin and gentamicin. The mutant MaadA1 containing an inactivated *aadA1* gene, was sensitive to streptomycin and spectinomycin, while it was still resistant to rifampicin, kanamycin, tobramycin, netilmicin and gentamicin. These results indicated that the genes *aacA3*, *arr3*, and *aadA1* were required for the resistance to kanamycin, tobramycin, netilmicin and gentamicin, rifampicin, streptomycin and spectinomycin, respectively.

### Function Confirmation of the Gene *aacA3*, *arr3* and *aadA1* in Gene Cassettes

PCR amplification for the genes *aacA3, arr3* and *aadA1* and a segment of integron containing all three genes *aacA3*, *arr3*, *aadA1*, generated a 626-bp, 553-bp, 863-bp and 2389-bp DNA fragment respectively (Table S1). Sequencing results showed the fragments obtained were the target genes and the enzyme sites were successfully introduced.

Four recombinant plasmids were constructed by ligating the four fragments to the vector pUFR034 and separately introduced into PXO99 by electroporation. After being harvested from NA plates with kanamycin, positive transformants were selected through PCR. PXO1-1, PXO2-1, PXO3-1 and PXO4-1 were four positive transformants, from plasmids of which expected target gene fragments about 626-bp, 553-bp, 863-bp and 2389-bp length were amplified (Figure S1). Double digestion assay confirmed that plasmids from positive transformants can be digested into two fragments by *Sac*I and *Kpn*I. The larger fragment was 8.7kb, exactly the length of pUFR034, and the smaller fragment was the same length as the ligated gene.

The MIC determination result showed that all four positive transformants were resistant to kanamycin corresponding to the vector pUFR034; PXO1-1 was resistant to gentamicin, tobramycin and netilmicin; PXO2-1 was resistant to rifampicin, PXO3-1 was resistant to streptomycin and spectinomycin; PXO4-1 was resistant to all the six antibiotics; and the resistance degree was as same as YNA11-2, except the streptomycin-resistance was slightly lower than YNA11-2 (Table 5). To sum up, PXO1-1 obtained the *accA3* gene, showing resistance to gentamicin, tobramycin and netilmicin; PXO2-1 obtained the *arr*3 gene, showing resistance to rifampicin; PXO3-1 obtained the *aadA1* gene, showing resistance to streptomycin and spectinomycin; and PXO4-1 obtained a segment of integron containing all three genes *aacA3*, *arr3*, *aadA1*, showing resistance to the six antibiotics, which confirmed the function of the genes *aacA3*, *arr3* and *aadA1* in gene cassettes.

### Stability of the Resistance Integrons

The stability of the resistance integrons was assessed in the four integron-carrying isolates by growing the isolates on media without streptomycin and then assessing streptomycin resistance and determining whether the *intI1* gene and the *aadA1* gene were present by PCR. These results are listed in Table S2.

All transfers (the growth after each transfer on medium without streptomycin is considered a transfer) of YNA7-1 and YNA12-2 showed streptomycin resistance and retained the *aadA1* gene and the *intI1* gene, indicating that the integron was stable in YNA7-1 and YNA12-2 after antibiotic removal.

For YNA10-2 in the first trial, only 50% of the colonies were resistant in the 10th transfer but 100% of the colonies were resistant in the 20th transfer. The percentage of colonies containing the *intI1* gene was only 11% in the 10th transfer and 0% in the 20th transfer. In the second trial, 90% of the colonies were resistant in the 5th transfer, and resistance percentage remained between 90 and 95% in the 10th, 15th and 20th transfer. The percentage of colonies containing the *intI1* gene in the second trial was only 5% in the 5th transfer and was 0% by the 10th transfer. The results of the two trials indicated that, when grown in the absence of streptomycin, YNA10-2 had stable streptomycin resistance but quickly lost the *intI1* gene.

For YNA11-2 in the first trial, 29% of the colonies were resistant in the 10th transfer and 0% were resistant in the 20th transfer; the *intI1* gene was completely lost in the 10th and 20th transfer. In the second trial, 85% of the colonies were resistant in the 10th transfer but resistance percentage declined to 25% in the 20th transfer; the *intI1* gene was detected in all colonies in all transfers. Overall, with respect to the streptomycin resistance of YNA11-2 the results of both trials were consistent, i.e., the resistance was gradually lost in the absence of streptomycin. The two trials differed with respect to stability of the *intI1* gene in YNA11-2; the *intI1* gene was completely lost by the 10th transfer in the first trial but was detected in all colonies of all transfers in the second trial.

The above results showed that the stability of streptomycin resistance differed among YNA7-1, YNA10-2, YNA11-2, and YNA12-2 and the phenotype of streptomycin resistance was completely consistent with the presence of the *aadA1* gene in all tested isolates.

## Discussion

Integrons are natural gene-capture systems that play an important role in dissemination of multidrug resistance, especially in gram-negative bacteria [18], [42]. The present study indicated that the class 1 integrase gene *intI1* and the aminoglycoside adenylyltransferase gene *aadA1* were present in all four resistant isolates but not in 25 sensitive isolates. The tests for resistance stability also demonstrated that the phenotype of streptomycin resistance was completely consistent with the presence of the *aadA1* gene in all isolates tested. The mutagenesis in three resistance genes indicated that the *aacA3* gene, the *arr3* gene, and the *aadA1* gene were required for the resistance to kanamycin, tobramycin, netilmicin and gentamicin, rifampicin, streptomycin and spectinomycin, respectively. Complementation tests further indicated the genes *aacA3*, *arr3*, and *aadA1* conferred resistance to corresponding antibiotic. The above results confirmed that the *aadA1* gene, which is carried by the integron, confers resistance to streptomycin in field isolates of *X. oryzae* pv. *oryzae*.

Resistance integrons have been reported in many clinical pathogens of gram-negative bacteria [18], [22]. They also have been found in many genera of bacteria isolated from cattle, pigs, chickens, duck, dogs, and zoo animals [22]. Isolates carrying integrons occur not only among bacterial pathogens but also among bacteria from environmental samples, including river water, waste water, agricultural soil and manured soil, *et al*. [22]. Resistance integrons have also been detected in phylloplane bacteria (*Pseudomonas* spp.) in an apple orchard [30]. Although resistance integrons are ubiquitous in bacteria from a wide variety of sources, no resistance integron had been reported from any phytopathogenic bacteria before the current report. In present study, a class 1 resistance integron containing the *aadA1* gene cassette and other antibiotic resistance gene cassettes were found in *X. oryzae* pv. *oryzae*. To our knowledge, this is the first report of a resistance integron in a phytopathogenic bacteria.

Based on the data reported here, we can make some inferences regarding the origin of the resistance integrons in the four resistant isolates of *X. oryzae* pv. *oryzae*. Firstly, the nucleotide sequences of the four integrons were identical except for absent parts, indicating that the four integrons originated from the same source. This inference was also supported by sequences of the *aadA1* genes, which were identical in the four resistant isolates but had 99% identity with the most similar *aadA1* gene in the GenBank database. The difference among the nucleotide sequences of the four integrons, which was only absent parts, could be attributed to the instability of the integron. Secondly, the BLAST search revealed that no integron in GenBank had cassettes arrayed in the same order as those in the integrons in the four isolates of *X. oryzae* pv. *oryzae*. Although similar gene cassette arrays were scarce in GenBank, it is too early to speculate about the origin of the gene cassettes in the four resistant isolates of *X. oryzae* pv. *oryzae.* It is known that antibiotics other than streptomycin (such as rifampicin, tobramycin, gentamicin, netilmicin, and kanamycin) had not been used to control bacterial blight of rice, however streptomycin-resistant isolates contained, in addition to the *aadA1*, the gene *aacA3*, which confers resistance to tobramycin, gentamicin, netilmicin, and kanamycin, and the gene *arr3*, which confers resistance to rifampicin. It is reasonable to speculate that the *aacA3* gene cassette and the *arr3* gene cassette might be simultaneously transferred together with the *aadA1* gene cassette to *X. oryzae* pv. *oryzae* when the bacteria were under the selective pressure of streptomycin. Therefore, the resistance integron of the phytopathogen *X. oryzae* pv. *oryzae* might originate from pathogenic bacteria from humans or animals treated with antibiotics such as tobramycin, gentamicin, netilmicin, kanamycin, or rifampicin.

Gillings *et al.* reported chromosomal integrons existed in *Xanthomonas* [43]. To understand the relationship between chromosomal integrons and mobile inegrons in *X. oryzae* pv. *oryzae*, the 3250-bp nucleotide sequences containing the chromosomal *intIA* gene and its flanking region were amplified from the four resistant isolates and two sensitive isolates. The result showed that the amplified nucleotide sequences of resistant isolates and sensitive isolates were 100% identical. Though chromosomal integrons (superintegrons) are considered to be the ancestor of mobile integrons and the resistance gene cassettes according to experimental and phylogenetic data [20], the *intI1* gene in resistance integron in *X. oryzae* pv. *oryzae* was 100% identical with *intI1* genes, which have been found extensively in many Gram-negative bacteria, the *intI1* gene and the whole resistance integron in resistant isolates of *X. oryzae* pv. *oryzae* should be derived from recent horizontal transfer event. Cambray *et al.* recently conducted a systematic study on available integrases [44]. The results showed a significant correlation between the loss of LexA regulation and integrase inactivation and the loss of LexA regulation seemed to be normal among soil and freshwater species harboring chromosomal integrons including Xanthomonadaceae, it meant that the chromosomal integrase in *X. oryzae* pv. *oryzae* may be inactivated. Therefore it is believed that there was no direct relationship between the chromosomal integrase and the resistance integron in *X. oryzae* pv. *oryzae*.

If resistance was always stable when the antibiotic pressure was absent, the risk of resistance development would be high. However, the resistance stability differed among the four integron-carrying isolates, which complicates the assessment of resistance risk. The high stability of the integron in YNA12-2 might be attributed to its incomplete integrase gene, which resulted in the non-function of integrase to integrate or excise gene cassettes; consequently, the *aadA1* gene cassette was persistent and resistance was stable. The reason for the difference of stability in the other three isolates is unknown. For the sequences of integrons in the isolates YNA7-1 and YNA11-2 were 100% identical, and the unique sequence difference between YNA10-2 and YNA7-1 was the deletion of the *aacA3* gene, so the difference of stability was not related to the sequence itself but probably attributed to its genetic location. The resistance integron, which is also referred as mobile integron, is usually linked to a mobile DNA element, such as a plasmid or transposon [20], but in our study the result of extraction of plasmid was uncertain (data not shown), which meant we have not determined the genetic locations of the integrons in resistant isolates of *X. oryzae* pv. *oryzae* to date. Another interesting phenomenon was the loss of the *intI1* gene, not only in YNA12-2 but also in descendants of YNA10-2 and YNA11-2. The loss of integrase gene, which is not universal in integron, is probably also related to the genetic location of integron.

The pathogenicity of the four field isolates of *X. oryzae* pv. *oryzae*, which carried the *aadA1* gene cassette on the integron conferring resistance to streptomycin, was not affected by the streptomycin resistance [11], and the stability of resistance differed among these isolates. Further studies on fitness, including growth rate and competitive ability, are needed to assess the risk of resistance.

In addition to being associated with resistance to multiple antibiotics, integrons also play a significant role in the dissemination of antibiotic resistance because of their abilities to integrate gene cassettes [45]. It is therefore possible that *X. oryzae* pv. *oryzae* and other phytopathogen may be a new source of resistance to multiple antibiotics in the future. Conjugation assays between homologous and heterologous bacterial species should be conducted. In china the ratio of streptomycin-resistant isolates containing the *aadA1* gene on the integron was very low (0.75%, 4/534) in the field [11], however, for the sampling area of Yunnan Province the percentage of resistance isolates is much higher (26.7%, 4/15). So the risk of streptomycin resistance in *X. oryzae* pv. *oryzae* and the risk of resistance integron spread through phytopathogen need more investigation and research to provide more evidence.

## Supporting Information

Figure S1 **PCR confirmation of recombinant plasmids from transformants.** M, DL2000 Marker; lane 1–4, plasmid pUFR034, amplified by primer pairs aac6f/aac6r, arr3/arr3r, aadaf/aadar, and integronf/integronr, respectively; lane 5, plasmid from PXO1-1, amplified by aac6f and aac6r; lane 6, plasmid from PXO2-1, amplified by arr3f and arr3r; lane 7, plasmid from PXO3-1, amplified by aadaf and aadar; lane 8, plasmid from PXO4-1, amplified by integronf and integronr.
(TIF)

Table S1 **Primer pairs used in this study.**
(DOCX)

Table S2 **Stability of streptomycin-resistance phenotype and the** ***aadA1*** **and** ***intI1*** **gene in resistant isolates of** ***X. oryzae*** **pv.** ***oxyzae*** **in the absence of streptomycin.**
(DOCX)
