# Supplementary figures and images for: Identification and Characterization of Integron-Mediated Antibiotic Resistance in the Phytopathogen Xanthomonas oryzae pv. oryzae
Source: PLoS One. 2013 Feb 21;8(2):e55962. doi: 10.1371/journal.pone.0055962 (PMC3578876; doi:10.1371/journal.pone.0055962)

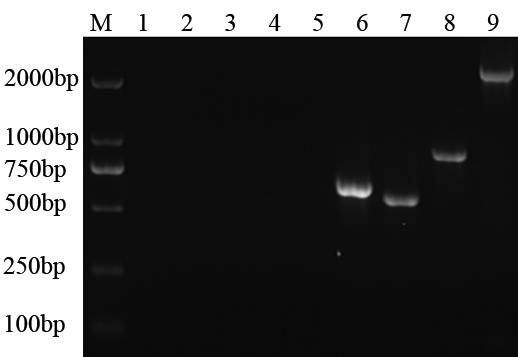

Supplement: Figure S1 — PCR confirmation of recombinant plasmids from transformants. M, DL2000 Marker; lane 1–4, plasmid pUFR034, amplified by primer pairs aac6f/aac6r, arr3/arr3r, aadaf/aadar, and integronf/integronr, respectively; lane 5, plasmid from PXO1-1, amplified by aac6f and aac6r; lane 6, plasmid from PXO2-1, amplified by arr3f and arr3r; lane 7, plasmid from PXO3-1, amplified by aadaf and aadar; lane 8, plasmid from PXO4-1, amplified by integronf and integronr. (TIF) [file pone.0055962.s001.tif]
