# Supplementary material for: Identification and Characterization of Integron-Mediated Antibiotic Resistance in the Phytopathogen Xanthomonas oryzae pv. oryzae
Source: PLoS One. 2013 Feb 21;8(2):e55962. doi: 10.1371/journal.pone.0055962 (PMC3578876; doi:10.1371/journal.pone.0055962)
Supplement: Table S1 — Primer pairs used in this study. (DOCX) [file pone.0055962.s002.docx]

**Table S1.** Primer pairs used in this study

| **Primer** | **Oligonucleotide primer (5'→ 3') or reference** | **Expected PCR product** | **Conditions of PCR** |
| --- | --- | --- | --- |
| Int1.F | [31] | 484-bp *intI1* gene fragment | 94°C 5 min; 30 cycles of 94°C 1 min, 60°C 1 min, and 72°C 1 min; 72°C 10 min |
| intI-R (Int1.R) | [31] |  |  |
| Int2.F | [31] | 789-bp *intI2* gene fragment | As amplifying with primer pair Int1.F and intI-R |
| Int2.R | [31] |  |  |
| Int3.F | [31] | 980-bp *intI3* gene fragment | As amplifying with primer pair Int1.F and intI-R |
| Int3.R | [31] |  |  |
| aadA.F | [31] | 743-bp *aadA1* gene fragment | 94°C 5 min; 30 cycles of 94°C 1 min, 67°C 1 min, and 72°C 1 min; 72°C 10 min |
| aadA1-R (aadA.R) | [31] |  |  |
| aadA2-S | CGCCATCTGGAATCAACG | 521-bp *aadA2* gene fragment | As amplifying with primer pair Int1.F and intI-R |
| aadA2-A | TACCAAATGCGGGACAAC |  |  |
| aadA1-R (aadA.R) | [31] | To characterize 5' CS of the integron | 94°C 5 min; 30 cycles of 94°C 1 min, 67°C 1 min, and 72°C 3 min; 72°C 10 min |
| IRI | [31] |  |  |
| intI-R (Int1.R) | [31] | To characterize 3' CS of the integron | 94°C 5 min; 35 cycles of 94°C 1 min, gradient temperature 50-62°C 1 min, and 72°C 3 min; 72°C 10 min |
| IRT | [31] |  |  |
| qac.F | [31] | 274-bp *qac△E1* gene fragment | 94°C 5 min; 35 cycles of 94°C 1 min, gradient temperature 50-62°C 1 min, and 72°C 1 min; 72°C 10 min |
| qac.R | [31] |  |  |
| sul.F | [31] | 790-bp *sul1* gene fragment | As amplifying with primer pair qac.F and qac.R |
| sul.R | [31] |  |  |
| TniR-a | GTTCGAGCGTGAGTTGATTTCC | About 4-kb sequence of integron | 94°C 5 min; 35 cycles of 94°C 1 min, 66°C 1 min, and 72°C 4 min; 72°C 10 min |
| IRI | [31] |  |  |
| TniA | GCCAGTCCGGTGGAGGTAAAGGTA | About 4-kb *tni* module | 95°C 5 min; 35 cycles of 94°C 30 s, 65°C 30 s, 72°C 3 min and 30 s; 72°C 10 min |
| TniaR | TGTTCGCTCGGAAATCAACTCACG |  |  |
| ilvd-f | GGATAAGGCGTTGTTGGA | 3250-bp *intIA* gene | 95°C 5 min; 30 cycles of 94°C 30 s, 55°C 30 s, and 72°C 2.5 min; 72°C 10 min |
| intergon-r | GAGTTGTTAGGCAGTGAGT |  |  |
| aac6f | ATAGAGCTCAAACAAAGTTAGGCAGCACAG | 626-bp *aacA3* gene | 94°C 5 min; 30 cycles of 94°C 30 s, 55°C 30 s and 72°C 30 s; 72°C 5 min |
| aac6r | CGGGGTACCGCGTCGGCTTGAATGAG |  |  |
| arr3f | ATAGAGCTCCGCGGCTTAATTCAGGTGT | 553-bp *arr*3 gene | As amplifying with primer pair aac6f and aac6r |
| arr3r | CGGGGTACCCGGTTTGATGGCTTTGTTA |  |  |
| aadaf | ATAGAGCTCACCCTGTCGCCGTTTAT | 863-bp *aadA*1 gene | As amplifying with primer pair aac6f and aac6r |
| aadar | CGGGGTACCCTTGAACGAATTGTTAGACAT |  |  |
| integronf | ATAGAGCTCGGATGAAGGCACGAAC | 2389-bp for integron fragment containing *aac*A3, *arr*3 and *aadA*1 | 95°C 5 min; 30 cycles of 94°C 30 s, 50°C 30 s and 72°C 30 s; 72°C 10 min |
| integronr | CGGGGTACCGCTGTCGTTCAACGCTTG |  |  |
| MaacA3-S | AGCGACCGACTCTTGATG | 348-bp *aacA3* gene fragment | 94°C 5 min; 30 cycles of 94°C 30 s, 57°C 1 min, and 72°C 1 min; 72°C 10 min |
| MaacA3-A | CGAATCCTGCCTTCTCATA |  |  |
| Marr3-S | TACAAGCAGGTGCAAGGA | 324-bp *arr3* gene fragment | As amplifying with primer pair MaacA3-S and MaacA3-A |
| Marr3-A | TTCAACAACGCCAACAAT |  |  |
| MaadA1-S | CTGGAGGGAGCGAGATTC | 355-bp *aadA1* gene fragment | As amplifying with primer pair MaacA3-S and MaacA3-A |
| MaadA1-A | TACCAAATGCGGGACAAC |  |  |
| accyan1-S | CGTGGAAACGGATGAAGG | 1355-bp fragment | 94°C 5 min; 30 cycles of 94°C 30 s, 57°C 1 min, and 72°C 1 min; 72°C 10 min |
| accyan1-A | CAGGGTCGGAACAGGAGA |  |  |
| accyan2-S | GGTATGTAGGCGGTGCTA | 2323-bp fragment | 94°C 5 min; 30 cycles of 94°C 1 min, 59°C 1 min, and 72°C 2 min; 72°C 10 min |
| accyan2-A | TCGGCTTGAATGAGTTGT |  |  |
| arryan2-S | TCGGTGATGACGGTGAAA | 941-bp fragment | 94°C 5 min; 30 cycles of 94°C 30 s, 55°C 1 min, and 72°C 1 min; 72°C 10 min |
| arryan2-A | ATAAACGGCGACAGGGTG |  |  |
| arryan4-S | GCTGGTGGGAAGATGAAA | 1329-bp fragment | As amplifying with primer pair arryan2-S and arryan2-A |
| arryan4-A | CAGGGTCGGAACAGGAGA |  |  |
| aadayan4-S | CGAGGACGGTCGTATTCT | 1164-bp fragment | As amplifying with primer pair accyan1-S and accyan1-A |
| aadayan4-A | CAGGCTTTACACTTTATGCTTC |  |  |
| aadayan5-S | AGCATTTATCAGGGTTATTGTC | 1429-bp fragment | As amplifying with primer pair arryan2-S and arryan2-A |
| aadayan5-A | TGCGAAGAACTCGGGATT |  |  |

Primer characters underlined are introduced restriction enzyme site. ‘GAGCTC’ is the recognition sequence of [restriction](app:ds:restriction) [enzyme](app:ds:enzyme) *Sac*I*,* ‘GGTACC’ is the recognition sequence of *Kpn*I. Characters in shadows are protective bases.
