# Supplementary material for: Identification and Characterization of Integron-Mediated Antibiotic Resistance in the Phytopathogen Xanthomonas oryzae pv. oryzae
Source: PLoS One. 2013 Feb 21;8(2):e55962. doi: 10.1371/journal.pone.0055962 (PMC3578876; doi:10.1371/journal.pone.0055962)
Supplement: Table S2 — Stability of streptomycin-resistance phenotype and the aadA1 and intI1 gene in resistant isolates of X. oryzae pv. oxyzae in the absence of streptomycin. (DOCX) [file pone.0055962.s003.docx]

**Table S2.** Stability of streptomycin-resistance phenotype and the *aadA1* and *intI1* gene in resistant isolates of *X. oryzae* pv. *oxyzae* in the absence of streptomycin

| **Isolate** | **Trial** | **Transfer^a^** | **No. of tested colonies** | **Resistance phenotype** | | ***aadA1* gene** | | ***intI1* gene** | |
| --- | --- | --- | --- | --- | --- | --- | --- | --- | --- |
|  |  |  |  | **No. of positive colonies** | **Percentage (%)** | **No. of positive colonies** | **Percentage (%)** | **No. of positive colonies** | **Percentage (%)** |
| YNA7-1 | 1 | 10th | 17 | 17 | 100 | 17 | 100 | 17 | 100 |
|  |  | 20th | 20 | 20 | 100 | 20 | 100 | 20 | 100 |
|  | 2 | 1st | 20 | 20 | 100 | 20 | 100 | 20 | 100 |
|  |  | 5th | 20 | 20 | 100 | 20 | 100 | 20 | 100 |
|  |  | 10th | 20 | 20 | 100 | 20 | 100 | 20 | 100 |
|  |  | 15th | 20 | 20 | 100 | 20 | 100 | 20 | 100 |
|  |  | 20th | 20 | 20 | 100 | 20 | 100 | 20 | 100 |
| YNA10-2 | 1 | 10th | 18 | 9 | 50 | 9 | 50 | 2 | 11 |
|  |  | 20th | 21 | 21 | 100 | 21 | 100 | 0 | 0 |
|  | 2 | 1st | 20 | 20 | 100 | 20 | 100 | 20 | 100 |
|  |  | 5th | 20 | 18 | 90 | 18 | 90 | 1 | 5 |
|  |  | 10th | 20 | 19 | 95 | 19 | 95 | 0 | 0 |
|  |  | 15th | 20 | 18 | 90 | 18 | 90 | 0 | 0 |
|  |  | 20th | 20 | 19 | 95 | 19 | 95 | 0 | 0 |
| YNA11-2 | 1 | 10th | 14 | 4 | 29 | 4 | 29 | 0 | 0 |
|  |  | 20th | 21 | 0 | 0 | 0 | 0 | 0 | 0 |
|  | 2 | 1st | 20 | 20 | 100 | 20 | 100 | 20 | 100 |
|  |  | 5th | 20 | 20 | 100 | 20 | 100 | 20 | 100 |
|  |  | 10th | 20 | 17 | 85 | 17 | 85 | 20 | 100 |
|  |  | 15th | 20 | 15 | 75 | 15 | 75 | 20 | 100 |
|  |  | 20th | 20 | 5 | 25 | 5 | 25 | 20 | 100 |
| YNA12-2 | 1 | 10th | 16 | 16 | 100 | 16 | 100 | 16 | 100 |
|  |  | 20th | 21 | 21 | 100 | 21 | 100 | 21 | 100 |

^a^Isolates grew for 2 days on NA plate without streptomycin (1st transfer) and were transferred to the other fresh NA plate without streptomycin (2nd transfer) until 20 transfers.
